# Prioritizing long-acting injectable antiretroviral therapy among key populations: Perceptions of persons living with HIV who inject drugs, ART clinic staff, and policymakers in Vietnam

**DOI:** 10.1371/journal.pone.0325195

**Published:** 2025-06-11

**Authors:** Minh X. Nguyen, Vivian F. Go, Adams L. Sibley, Hanna E. Huffstetler, Trang N.T. Do, Ha V. Tran, Le M. Giang, Teerada Sripaipan, William C. Miller, Joseph J. Eron, Cynthia L. Gay, Sarah E. Rutstein

**Affiliations:** 1 UNC Project Vietnam, Hanoi, Vietnam; 2 Department of Epidemiology, Institute of Preventive Medicine and Public Health, Hanoi Medical University, Hanoi, Vietnam; 3 Department of Health Behavior, Gillings School of Global Public Health, University of North Carolina, Chapel Hill, North Carolina, United States of America; 4 Center for Research and Training on Substance Use and HIV (CREATA-H), Hanoi Medical University, Hanoi, Vietnam; 5 Department of Epidemiology, Gillings School of Global Public Health, University of North Carolina, Chapel Hill, North Carolina, United States of America; 6 Division of Infectious Diseases, Department of Medicine, University of North Carolina, Chapel Hill, North Carolina, United States of America; Riphah International University, PAKISTAN

## Abstract

**Objectives:**

We explored perceptions of long-acting injectable (LAI) patient prioritization and decision-making among people who inject drugs (PWID) with HIV, HIV clinic staff, and policymakers in Vietnam.

**Methods:**

From February to November 2021, we conducted 38 interviews with 19 PWID, 14 HIV clinic staff, and 5 policymakers in Hanoi, Vietnam. Interviews were coded and analyzed using thematic analysis to assess themes across participants.

**Results:**

PWID highlighted the importance of medical providers in treatment decisions, while clinic staff stressed adequate counseling and patient choice. Non-patient participants viewed adherence to Ministry of Health guidelines as essential, and many clinic staff saw PWID as ideal for LAI due to suboptimal adherence. Policymakers, however, preferred prioritizing LAI based on viral suppression and adherence rather than risk behaviors.

**Conclusions:**

This study reveals conflicting stakeholder views on LAI prioritization, suggesting a need for a more nuanced implementation approach to balance efficiency and fairness in low-resource settings.

## Introduction

The Joint United Nations Programme on HIV/AIDS (UNAIDS) has widely promoted the goal of ending the HIV/AIDS epidemic by 2030 [1]. To help achieve this goal, antiretroviral therapy (ART) modalities that do not require daily dosing might be an important strategy to improve viral suppression and maximize treatment outcomes for persons with HIV (PWH) who struggle to adhere to daily oral ART [2,3]. Long-acting injectable (LAI) ART is a novel alternative to daily oral pills [4]. The combination of once monthly or every-two-months long-acting cabotegravir and long-acting rilpivirine has been approved for use in the United States and Europe as treatment for adolescents and adults living with HIV who are virally suppressed (HIV RNA < 50 copies/ml) [3,5,6], and registration processes are ongoing in other parts of the world [7]. Findings from phase 3 clinical trials found that LAI ART (hereinafter referred to as “LAI”) is non-inferior to daily oral ART [8,9]. This new modality holds great potential to revolutionize treatment for millions of PWH by decreasing the daily pill burden from 365 days to only a few injections per year [4]. LAI may reduce treatment fatigue due to high pill burden, improve adherence, and reduce HIV-related stigma due to its discreet treatment [10–12].

LAI is more expensive to produce and deliver relative to oral treatments. Consequently, cost is an important structural barrier to widespread LAI use [12,13]. The high costs of LAI may make it essential to prioritize LAI for populations who will benefit the most from this regimen, particularly in more resource-constrained settings. So far, phase 3 LAI trials have been restricted to PWH with good adherence to daily oral ART, evidenced by viral suppression [8,9]. But LAI appears to be cost-effective if explicitly targeted to PWH who would be expected to have viral failure on oral ART due to inadequate adherence [14]. Hence, scale-up of LAI will introduce novel decision-making considerations among policy makers, clinicians, and patients.

As LAI becomes approved for use, careful preparation is needed to introduce LAI efficiently, equitably, and acceptably, especially in low resource settings where availability of LAI may be limited. Yet the attitudes of stakeholders and PWH regarding priorities for LAI use are unknown and it is unclear whether and how the implementation of LAI should be regulated with these considerations in mind. For example, should LAI be prioritized for PWH with predicted challenges to viral suppression or only those with demonstrated failure? And who gets to decide whether LAI is right for a particular person? Among people who might benefit from LAI, people who inject drugs (PWID) living with HIV are unique. PWID often struggle with adherence and might benefit from regimens that do not require daily adherence [15]. Indeed, injection drug use is a strong risk factor for suboptimal ART adherence globally [15–17]. But PWID are frequently excluded from LAI clinical trials. As a result, the appropriate way to consider injection drug use behaviors in LAI decision-making is unclear.

In Vietnam, PWID are the predominant group of PWH [18]. Vietnam is also one of six countries accounting for half of the global population of PWID, and the HIV prevalence among Vietnamese PWID is 29.3% [19]. Vietnamese PWID with HIV may benefit from LAI, but preferences and demand are not known. Although LAI has not yet been approved for HIV treatment in Vietnam, approval is expected soon. We conducted a qualitative study among PWID with HIV, ART clinicians, and policymakers in Vietnam to explore their perceptions on patient prioritization and decision-making for LAI.

## Methods

### Study setting and population

This study was conducted in Hanoi – the capital of Vietnam and its second largest city. The prevalence of HIV among PWID in Hanoi is among the highest in the country [18]. From February to November 2021, we invited policymakers, ART clinic staff (physicians, counselors, and pharmacists), and PWID with HIV in Hanoi, Vietnam to participate in brief surveys and in-depth interviews (IDIs). Full details of the study procedures are described elsewhere [12].

Policymakers and ART clinic staff were eligible to participate if they were at least 18 years old and were working in HIV/AIDS government agencies or public ART clinics, respectively. Eligibility criteria for PWID included being at least 18 years old, injection drug use in the past 6 months, and being diagnosed with HIV. We purposively recruited PWID from all three groups: those who were either enrolled in care, ART-naïve, or enrolled in ART care but already dropped out. Participants were recruited through outreach within professional established networks at HIV/AIDS government agencies and four public ART clinics with co-located substance use disorder treatment services. A pre-determined sample size was determined based on that which we anticipated would yield sufficient results recognizing the scope of our inquiry and the budget constraints therein. Saturation was determined by our reviewing transcripts and reaching the consensus that we were not getting substantially different responses from the participant groups.

### Data collection

All participants provided written consent in a private setting. After informed consent, enrolled participants completed a brief sociodemographic survey prior to the IDIs. IDIs were conducted in private rooms at the clinics by a trained Vietnamese interviewer to ensure privacy (who was not included as an author in the paper). The interviewer had extensive experience conducting IDIs with PWH and healthcare staff in Vietnam before. Interviews with PWID and ART providers were conducted within the ART or substance use disorder treatment clinic; policymaker interviews were conducted at a location of their choosing, typically private offices. The interviews were audio-recorded, transcribed, and translated into English. Semi-structured interview guides were developed by the research team. These guides was developed based on the Consolidated Framework for Implementation Research (CFIR) framework. After querying respondents about their familiarity with LAI, all interviews included a brief introduction and background on LAI, including dosing frequency and efficacy data from randomized controlled trials. IDIs further explored participants’ experience with ART (including prescribing and program development or implementation), perceptions on patient groups that may benefit the most from LAI, and factors that can influence the decision of clinicians to prescribe and of patients to receive LAI. For PWID, we asked questions to understand how they would make the choice between daily oral or monthly injectable ART, if both were available. For clinic staff, we explored how they would decide when and whom to offer LAI and their opinions on PWH’s option to choose between daily pills and LAI. For policymakers, we asked questions on who should be offered LAI were it to be approved for use in Vietnam and explored their opinions on policies that prioritized PWH based on HIV risk behaviors or those who were lost to care. We worked with the Vietnamese members of the research team and the local community to confirm translation, understanding of each question in local language, and refine the interview guides. In-depth interview guides are included in the Supplementary S1 File.

After each IDI, participants received compensation for their time and travel. The study received ethical approval from the Institutional Review Boards at the University of North Carolina at Chapel Hill (UNC) and Hanoi Medical University.

### Data analysis

Survey responses were summarized using descriptive statistics (Stata version 13.1, College Station, Texas, USA). All interview transcripts were imported into Dedoose (version 7.0.23, Los Angeles, CA, SocioCultural Research Consultants, LLC) for the purpose of coding and data analysis. Interviews were coded and analyzed using thematic analysis to assess themes across types of participants. A codebook was developed based on the main topics explored in the interview guides (deductive codes) and common themes across all interviews (inductive codes). Separate codebooks were developed for patient and non-patient (i.e., provider and policymaker) participants. One investigator independently coded all interview transcripts, and to ensure intercoder reliability, three co-investigators co-coded three transcripts and discussed any discrepancies. Memos and notes of emergent themes and patterns were written for each interview. During the data collection and data analysis phases, prioritization and patient selection emerged on as important themes and were focused on as the primary topic of this paper. Study results were generated after reviewing code reports from Dedoose alongside memos. We structured the results based on key themes captured by the codebook.

We adhere to the Consolidated Criteria for Reporting Qualitative Research (COREQ) checklist for qualitative research (Supplementary S2 File). Additional information regarding the ethical, cultural, and scientific considerations specific to inclusivity in global research is included in the Supplementary S3 File (Questionnaire on inclusivity in global research).

### Research team and reflexivity

The authors of this study bring extensive multidisciplinary expertise to this study, with advanced degrees in medicine, public health and pharmacy (MD, PhD, MPH, PharmD). Collectively, they have significant experience in clinical medicine, epidemiological research, health behavior, and infectious diseases, contributing a robust and comprehensive perspective to the study. Two thirds of the authors are female. Some members of the team in Vietnam were introduced to policymaker participants during the recruitment process. Neither the interviewer nor the authors had prior relationships with any participants before the study.

## Results

All invited participants agreed to participate. In sum, we conducted 38 IDIs with three groups of stakeholders (19 PWID with HIV, 14 ART clinic staff, and 5 HIV/AIDS policymakers). Among PWID with HIV, 3 (16%) were ART naïve, 14 (74%) were currently on ART and 2 (11%) had dropped out of care. The mean age of the PWID participants was 41 years old (range 34–47); 90% were male. The mean duration of time since HIV diagnosis was 11 years. ART clinic staff included four (29%) prescribing physicians, three (21%) counselors, three (21%) pharmacists, and four (29%) clinic directors. Among 5 policymakers, 2 were staff of the Vietnam Administrative for AIDS Control, and 3 worked at the Hanoi Department of Health or Centers for Disease Control. The IDIs lasted between 45 and 60 minutes.

### Perceptions of PWID

#### Preference for LAI.

All except three PWID participants stated that they would prefer LAI over daily pills. The main reasons given were that when compared to oral ART, LAI was more convenient and easier to adhere to because participants would not need to take or remember to take medication every day. The remaining participants acknowledged that LAI might be more convenient than oral ART, but if given the choice, they would want to keep taking oral medications. Two were hesitant because they did not know much about LAI yet and would need to wait for others to take it first; one reported no problems with daily pills.

“*I haven’t decided yet so I won’t try it. I have to see people use it for a few months before I dare to decide*.” – PWID, ID 113, currently on ART

#### Deference to medical authority.

PWID participants’ deference to medical authority was a salient theme across interviews. Many PWID stressed the important role of medical providers in their decision to get ART and choose the type of treatment modality. Participants deeply trusted their medical providers, feeling reassured that if they listened to their doctors’ counseling, they would not need to worry about anything. A few participants indicated that they would follow whatever their doctors told them to do. This commitment to follow their physician’s recommendations was motivated by trust, but also a belief that they did not have the right to choose, with one participant describing themselves as like “a fish on a chopping board”.

“*I think when I’m on treatment I will listen to doctors’ indications, so now I don’t know how the treatment is to think about it. I think I will follow doctors’ advice, if they counsel me on which medication is good, I’ll use it. I also don’t have the right to choose*.” – PWID, ID 102, ART naive

### Perceptions of ART clinic staff and policymakers

#### Freedom to choose.

All except two clinic staff and policymakers had heard of LAI for HIV treatment prior to the interviews. The most common sources of information were from colleagues and media (e.g., TV or newspaper). Most ART clinic staff liked the idea of giving PWID with HIV the power to choose an ART regimen for themselves. Clinic staff believed that PWH would embrace more options for treatment, rather than having to accept oral ART as the only choice. Respondents described that if clients got to pick the option that would be most appropriate for them, they would be happier, more motivated to get treatment, more responsible with their own choice, and more likely to adhere to their chosen regimen. Clinic staff explained that if the patients’ first choice resulted in poor adherence, clients could easily switch to another regimen.

*“Well, if so, I think clients are our God, we offer services and they will choose which service is right for them. That’s also a very good point… They will be more responsible, as they have been provided with information, and now they choose to use the injectable treatment, and for example, during treatment, they will have pains, they already know it themselves, so when they decide to take this injectable treatment, they know that they’re going to have pains, and they have to come here every month. I mean, they choose by themselves, so they will be more responsible.” –* Clinic staff, ID 204, Physician

Many clinic staff emphasized the importance of providing adequate counseling and letting clients make their own choice instead of deciding for them. Participants explained that it would be difficult for providers to guess which type of treatment their clients preferred, and because each client might have different barriers to adherence and staying in care. In general, ART clinic staff described that though LAI might work well for one client, it might not be suitable for another.

“*Well, actually, I can’t really prescribe treatment options for them, I can only give my clients options, and by that way, I feel really secure for doctors and medical staff who are providing ARV [antiretroviral] treatment for the patients. When we decide the treatment option for patients, it will be different from when we offer treatment options and let them choose. That is, it is the voluntary choice of the clients, so I think it’s very good. Firstly, for the treatment provider, it brings a much more secure feeling, secondly, the clients also feel more comfortable and favorable toward the clinic because they are given 2 options by the clinic, they are clearly explained the benefits and limitations so that they can make the decisions on their own.” –* Clinic staff, ID 206, Physician

#### Factors to consider when prioritizing LAI.

Although clinic staff generally agreed that good counseling based on evidence was necessary to help clients make their own choice, they had different, or even contrasting, opinions about which client groups would benefit from LAI. Nearly all clinic staff noted that clients who struggled to adhere to ART would benefit the most from LAI:

*“If we implement this service [LAI], patients will be so happy because they don’t need to remember anything. But do they need to be injected on time? Probably not, right? I think they only need to receive injections on the scheduled day, that’s fine. If that’s right, there’s no problem. Now they have to remember to take medicine, we also advise them to take medicine on time, but they still forget. For example, they go to their friends’ birthday parties and drink alcohol, so they don’t remember to take medicine, and when they remember, they don’t have medicine on hand to take.” –* Clinic staff, ID 203, Counselor

However, one participant took an opposite stance, stating that he would not offer LAI to those who did not comply well to their oral ART regimen.

“*It depends on their adherence, if patients comply well, they will remember the scheduled [injection] date; if they don’t adhere well to the treatment, I will not advise them to get injections. Because the frequency of injections is once a month, if the patient does not comply, they will not remember that day to come for injections. So I’m worried that those who do not comply will not remember the injection schedule. If I have to choose, I will choose people with good adherence to advise them on injections.” –* Clinic staff, ID 219, Counselor

Clinic staff further mentioned the occupation of clients as an important factor to consider when counseling on the choice between LAI and daily pills: those with demanding work schedules, those having difficulties taking a day off, those working in shifts and those traveling frequently or unexpectedly were perceived as good candidates for LAI. Between ART-naïve and ART-experienced but lost-to-care clients, some participants believed that ART-naïve participants would be more willing to receive LAI because these clients did not have much experience with oral pills to compare two options. In contrast, others considered LAI to be a more appealing choice for those who quit treatment due to adherence issues.

All clinic staff participants described PWID with HIV as a sub-population who frequently struggled with adherence. Many considered PWID an ideal population to receive LAI, emphasizing that LAI would not only help these clients adhere to ART, but also make it easier for providers to manage their patients and keep them in care, improving viral suppression for this difficult-to-suppress group. But some clinic staff argued that men who have sex with men (MSM) could have the same issues with adherence and prefer LAI, and therefore LAI should be offered to all groups and not be prioritized to anyone.

*“In my opinion, if injectable medicine is available, those who use drugs will be the first subjects that we will counsel to switch to the injectable type.” –* Clinic staff, ID 214, Physician

“…*MSM might like injections too. Maybe some MSM don’t take medicine on time due to their bad memories. I counseled many patients that they should take medicine on time, and they could only forget once in a month, but they said “Oh my God, I’ve forgotten to take medicine three times this month”, then they felt stressed or anxious or scared too much. So, I think it depends on clients’ needs, I don’t know who will want it*.” – Clinic staff, ID 203, Counselor

#### Policies regarding LAI prioritization.

In contrast to clinic staff, policymakers generally insisted that PWH should be treated equally regardless of which risk group they belonged to. They stressed that providers should be fair to all clients, only considering the viral suppression status or adherence to make recommendations on treatment modality, avoiding policies that explicitly prioritized PWID for eligibility to alternative treatment modalities. More specifically, they believed that those who failed to achieve viral suppression should be the priority group when it came to LAI:

*“I think so, so the problem here is no matter who the patients are, if they don’t achieve the viral suppression, we must care about them to give them the better and optimal treatment.”* – Policymaker, ID 208, Ministry of Health Official

In addition, one participant believed that focusing more on PWID was stigmatizing and discriminatory towards this group.

*“In terms of treatment, any patient in any group should have equality and counseling as well as the same treatment care. We shouldn’t classify patients into any group.” –* Policymaker, ID 209, CDC official

Aligning more with statements of ART clinic staff, two policymakers expressed interest in special treatment programs focusing on PWID, who might have unique needs and barriers to treatment that were different than other groups. In general, policymakers emphasized the success of existing ART programs in achieving viral suppression targets, even in the absence of LAI.

#### Dependence on guidelines from the Ministry of Health.

Although most ART clinic staff emphasized the importance of patient choice when it came to regimen selection, some ART clinic staff and policymakers mentioned the necessity of written guidelines from the Ministry of Health in helping providers decide when and to whom they could prescribe LAI. Closely following treatment guidelines from the Ministry of Health was perceived as mandatory, and this was felt to be particularly important with a new ART modality like LAI. One participant explained that since their care facilities were not research units, they would have little idea of whether a treatment was good or bad and would not know how it should be used to benefit ART clients, emphasizing the important distinction between clinical eligibility and prioritization based on other patient characteristics or criteria.

*“And to apply these regimens step by step, we completely follow the guidelines issued by the Department of HIV/AIDS Prevention and Control and the Ministry of Health, but we don’t research by ourselves, we are not a research unit to find out which regimens and medicine are good or not. We follow the guidelines of the Ministry of Health and its orientation.”* – Policymaker, ID 209, CDC official

## Discussion

With existing and anticipated approvals of LAI for HIV treatment globally, policymakers, providers and their patients may soon be able to choose between traditional oral regimens and newer LAI options. Unlike a new oral regimen, LAI represents a substantial divergence in terms of health system, clinic, and patient logistics, convenience, and cost. More frequent clinic visits, the potential pain of injections, evolving supply chains, and the expense of LAI all mean it is unlikely to immediately or completely replace oral ART, although many patients and providers desire an injectable therapy option [20–23].

Identifying suitable or “preferred” candidates for LAI is a major barrier to implementation at the provider level [13,24,25]. Providers from ART clinics have had mixed opinions on whether adherent or non-adherent patients would be preferred for LAI [24,25]. In this study, we shed light on perceptions regarding patient/provider interactions regarding treatment choice and the policy structures that may inform these options. We focused on PWID with HIV, a group that commonly has worse treatment outcomes [15,16,26]. From our findings, PWID tended to rely on medical authorities to decide for them when it came to HIV treatment. In contrast, HIV providers would prefer to let clients make their own choice. This “choice” was contextualized as having to depend on whatever the forthcoming written guidelines from the Vietnamese government outlined as appropriate criteria for LAI. Policymakers, on the other hand, emphasized the importance of equal access to HIV treatment, arguing that no specific predefined group of clients should be prioritized or receive more attention than others. These conflicting opinions seemingly complicate selecting candidates for LAI when it is taken to scale in Vietnam.

When it comes to LAI, cost is a critical consideration, and those participating in our study raised important questions about the financial components of introducing LAI as a treatment option. Although drug costs have not been determined in settings where LAI has not yet been registered, the regimen is expected to significantly exceed the cost of oral ART for the near future [27,28]. Even as drug costs decrease with more widespread LAI use, additional resources are expected with more frequent visits and other associated consumables. High cost is an important hurdle for LAI implementation in low- and middle-income countries (LMICs) [13], and prioritization of patients to receive LAI might be unavoidable. In our study, the issue of costs did not come up during the interviews as a factor to decide which groups should be prioritized to use LAI, but costs were identified as a substantial barrier and have been described in a previous publication [12]. The most cost-effective approach appears to be to offer LAI to the subgroup of PWH having difficulties achieving adequate adherence and viral suppression with daily oral ART [14,29]; this approach prioritizes the benefits of improving viral suppression. Although not explicitly modeled, this strategy would also be expected to result in reduced transmissions and avoid some of the costly transitions to second- and third-line oral ART regimens for persons who develop resistance due to inconsistent adherence. For example, although different scenarios for introduction of LAI could potentially avert disability-adjusted life year (DALYs), only the use of LAI among those with a viral load great than 1000 copies would be cost-effective [14]. A more complete understanding of which PWH will benefit most from LAI given different levels of adherence and viral suppression is urgently needed.

PWID may be one group that needs to be prioritized based on historically poor viral load outcomes, but some of our participants raised the concern that prioritizing them risks further stigmatizing this group. Moreover, the idea of prioritization was not fully embraced by policymakers in Vietnam. An alternative to group-based prioritization is making prioritization decisions based on clinical criteria, such as viremia, though the individual and public health implications of “waiting for viral failure” must be considered. Additional research is needed to identify culturally acceptable prioritization strategies that balance the potential stigma, individual, and public health benefits with the economic constraints of this more expensive treatment modality.

In many LMICs, ART adherence among PWID is below the minimum level required for viral suppression [30]. PWID in Vietnam have significantly poorer immune reconstitution and retention rates, compared to those without injection drug use [26]. Nonetheless, as participants explained in our study, even though the burden of daily pills might be alleviated for clients receiving LAI, these patients still must adhere to clinic appointments to receive monthly or every-two-month injections. Missing injection appointments might cause more serious consequences than missing an oral dose, with both individual and public health implications if persons fail with resistance to the common backbone integrase strand inhibitor [31]. Tracing systems, appointment reminders and integration into other services such as methadone maintenance treatment are possible solutions to overcome this issue of missed appointments.

In general, clinic staff in our sample supported the model of shared decision making, in which both medical providers and patients share information and decide together. Among PWH, shared decision making has been associated with better outcomes, such as higher satisfaction and better treatment adherence [32]. But shared decision making may not be embraced by all patients. The PWID with HIV in our study seemed to be more confident and familiar with a provider-driven model, during which patients passively consent to professional authority and agree to the doctor’s choice of treatment. They assumed that they have no power and doctors will make the best choice for them. Indeed, the healthcare context in Vietnam traditionally emphasizes hierarchal authority above provider-patient partnership [33]. PWID living with HIV in Vietnam may be unaccustomed to making decisions about their treatment modality because for their HIV and substance use treatment, daily oral ART and methadone maintenance treatment have historically been the only options offered [12,34]. In a multi-country Asian study among persons with cancer, most Vietnamese patients perceived themselves as having no role in the decision making process, and the proportion of patients in Vietnam who considered themselves being more involved in decision making than preferred was also the highest, when being compared with other Asian countries [33]. These findings might reflect socio-cultural norms related to medical care in lower income countries [33,35]. Healthcare providers should therefore engage patients in discussions to explore their decision-making expectations and provide care according to their preferences.

Among patients who favor shared decision making, patient decision aids could be used to facilitate the process [36]. As participants mentioned in the interviews, shifting from one regimen to another might be necessary during the treatment process. Decision tools that inform choices regarding initiation, discontinuation of LAI, as well as changing between LAI and oral ART would be important to help both clinicians and patients make the best choice. Recommendations on eligibility criteria, patient prioritization, risks and benefits of LAI at the global and national levels based on strong evidence would also be warranted to provide guidelines for clinicians and ensure consistent practice across countries and regions.

In this study, we were able to invite and conduct in-depth interviews with policymakers at the Vietnam Administrative for AIDS Control, Ministry of Health, who would be involved in changing and issuing HIV policies for the country. However, like most countries, the HIV policy-making process in Vietnam is complex, involving many agents including the Communist Party, the National Assembly and the Ministry of Health, and other national and international agencies with diverse objectives and priorities [37]. As such, our results reflect an important but narrow perspective of HIV policymakers who would be expected to influence guideline development and implementation.

Just as policymakers reflect diverse perspectives PWH occupy a similar range of lived experiences when it comes to ART use and preferences. Our recruitment strategy, using the clinics to identify PWID who were living with HIV, meant that we were frequently limited to those who were engaged in care. We did intentionally limit the number of PWH who were ART naïve as we assumed they had less experience with ART in general, and thus may have struggled to respond to questions regarding barriers or challenges with the standard of care oral ART. We did intentionally recruit PWH who had disengaged in care but, given our clinic-based recruitment, the vast majority (all but two) of PWH were engaged in care at the time of our interview. Perceptions of LAI for PWID living with HIV who were on ART but subsequently stopped may differ from those who are durably engaged in ART care, and are not well represented in the current study. In addition, the absence of viral suppression data for patient-participants, which may have provided additional context for interpreting their experiences and perspectives. Studies with a larger and more diverse sample of PWID with HIV and additional key informants from governmental and non-governmental agencies contributing to HIV management guideline development and programming, may provide important additional insights. Another limitation of this study is that the data were collected in 2021, and perceptions of long-acting injectable (LAI) HIV prevention and treatment may have evolved since then, particularly with the growing global experience and awareness of newer agents such as Lenacapavir. Future research is needed to capture how these views may shift over time as LAI options become more widely available and integrated into HIV care in low-resource settings. Despite this, our findings still provide valuable insights into the attitudes and considerations surrounding LAI at the time of data collection, which can inform ongoing and future research on patient preferences and implementation strategies.

## Conclusions

Our study provides unique and previously unexamined perspectives of LAI implementation and patient prioritization in an often overlooked key population of PWID living with HIV, through which we identify and expose critical complexities in the effort to balance equality and access with the reality of resource constraints.

Among PWID with HIV and HIV clinic personnel in Hanoi, Vietnam, LAI is a highly desirable treatment alternative to daily oral ART. Its unique advantages over oral ART may reduce barriers to viral suppression and facilitate achievement of the UNAIDS 95-95-95 goals [1]. But sustainable implementation, particularly in LMICs, will need to consider the resources associated with this novel treatment. The cost implications of LAI might create a challenging situation in LMICs, where demand is greater than supply, especially if many clinicians and PWH prefer LAI over daily pills. By exploring perceptions related to patient prioritization and selection among PWID with HIV, ART clinic staff, and policymakers in Vietnam, we identified implementation barriers related to the prescription of LAI by providers and uptake of LAI by PWID. We identified a need for a more nuanced approach to LAI implementation to ensure both efficiency and equality for PWH. Differential service delivery, shared decision making supplemented by decision aids, and guidelines for LAI prescription and patient selection may all be necessary to maximize treatment outcomes and bring this innovative treatment tool to scale.

## Supporting information

S1 File Qualitative interview guides: LAI_ART_Interview_Guide_27Apr_2021_combined.docx. (DOCX)

S2 File COREQ checklist for qualitative research: COREQ_LAI ART_4.26.25.docx. (DOCX)

S3 File Inclusivity in global research questionnaire: Inclusivity-in-global-research-questionnaire.docx. (DOCX)
