# Supplementary material for: Prioritizing long-acting injectable antiretroviral therapy among key populations: Perceptions of persons living with HIV who inject drugs, ART clinic staff, and policymakers in Vietnam
Source: PLoS One. 2025 Jun 11;20(6):e0325195. doi: 10.1371/journal.pone.0325195 (PMC12157207; doi:10.1371/journal.pone.0325195)
Supplement: S1 File — (DOCX) [file pone.0325195.s001.docx]

**Interview Guide**

Feasibility and Acceptability of Long-Acting Injectable Antiretroviral Therapy (LAI ART) among HIV-Infected People who Inject Drugs (PWID) in Vietnam

*(VERSION: PWID currently engaged in ART)*

**Purpose**: To characterize current access and adherence to daily oral ART and the feasibility and acceptability of long-acting injectable ART as an alternative.

**Population**: HIV-Infected PWID living in Hanoi or Khanh Hoa province.

**Introduction**: Hello and thank you for agreeing to speak with me today. My name is [NAME]. I am part of a research team seeking to improve treatment outcomes for people with HIV. As you may know, ART has traditionally been available from clinics as a daily oral tablet. More recently, scientists have developed an alternative form of ART available as a monthly injection. Research indicates that the monthly injection is as effective as the daily oral formulation in maintaining HIV viral suppression.

Today we would like to ask you about your experience as a person with HIV. We would like to understand your previous experiences accessing or trying to access treatment for HIV. We would also like to understand your thoughts and opinions about current treatment options. Finally, we would like to ask you your opinions about the monthly injectable form of ART. I would like to learn about these things from your perspective.

Anything that you say to me will be kept confidential. What we discuss is audio recorded and then transcribed. When it is written out, all information that could identify you to someone reading the words (anything said with names or locations or that kind of thing) are completely removed. The material from our interview is saved with your identification number but not your name. The research team members reviewing this text will not link who you are to that ID and will not tell anyone on site about your specific feedback. There are other people doing interviews as well, and we will put all those documents together and look at main topics people talked about. We will not identify or share anything you say as a comment linked to your name or ID number.

Your input is crucial to help us improve ART access in Vietnam. It is important for you to know that there are no right or wrong answers. If there are any questions that make you feel uncomfortable, feel free to tell me and we can skip or come back to those questions.

You have signed the informed consent form in which you consented to audio record this discussion. I may also be taking notes on the things you are saying to help me follow the discussion. Please recall that when these tapes are not being reviewed, they will be stored in a locked location and will be destroyed after analyses are completed.

I would like to start recording. Before we do this, do you have any questions or concerns?

Are you ready to begin?

**Introduction**

To start off, I’d like to learn a little more about you.

1. Can you walk me through a typical day in your life?

*Probes*: What kinds of routines do you have? (HINT: work, school, medical appointments, meals)

How do you like to spend your free time?
 Tell me about your job.

How long have you lived in this area?

1. Can you tell me about the important people in your life? This could be family, friends, people you work with – anyone who comes to mind.

*Probes*: Do you have family in this area?

Are you married? Have kids?

Who do you live with?

Who do you talk to when you need support, like a ride to the store?

What about people you use drugs with?

| RQ1. How do PWID currently describe accessing and adhering to ART? |
| --- |

Thank you for sharing with me. Next, I’d like to ask you a little bit about your experience living with HIV. I want to remind you again that everything you share is just between us. I won’t be sharing this information with your doctors or anyone else, so please feel free to talk honestly.

1. First, can you tell me how you came to learn that you have HIV?

*Probes*: Tell me all the things you remember about those few days.

Walk me through your diagnosis.

How did the medical staff treat you?

Did you disclose your status to anyone? Who did you tell first?

1. Can you walk me through the process of beginning ART?

*Probes*: About how long ago did you begin ART?

How did you decide to begin ART? Did anyone help you make this decision?

What did you see as the pros and cons of beginning ART?
 How did you feel about beginning ART? (HINT: Hopeful? Scared?)

Were there any times you thought about stopping ART?

1. Walk me through a typical visit to the ART clinic.

*Probes*: When you walk in the door, who’s the first person you see?
Who do you talk to or interact with?
How often do you go?

How do you get to the clinic? How far do you have to travel?

How long does an appointment take?

How long have you received care at this clinic?

Have you ever used a different clinic? (IF YES: How did your experience there compare with your experience here?)
Do you always come to the same clinic, or do you ever get your ART somewhere else? Why?

1. It’s common for people to experience challenges accessing healthcare. Can you tell me about what makes it difficult or easy to get your ART medication?

*Probes:* What, if any, are the challenges of getting to the clinic for testing?

What, if any, are the challenges of getting your prescription filled?

1. It’s also common for people to stop ART, whether for a day or two or for a long time. Can you tell me about anything that is difficult about staying engaged in ART?

*Probes:* Can you describe any times when you…

… Forgot to take your medicine?

… Didn’t have your medicine on hand, for example, because you were out of the home?

… Didn’t feel like you wanted to take your medicine? (What made you feel that way?)

… Stopped your treatment altogether, like for months or longer? How often has it happened?

Can you think of anything that would make it easier to take your medicine?

1. For many people, injection drug use can get in the way of day-to-day living. Can you think of any ways that your drug use makes it harder to take ART daily? (HINT: community/provider stigma, low motivation, lack of knowledge about ART, transportation/access/money issues)

*Probes*: …when you’re traveling to the clinic?

…when you’re talking to medical staff?

…when you’re at home with your family?

| RQ2. How do PWID describe the feasibility/acceptability of LAI ART, and how does this compare with their perceptions of daily oral ART? |
| --- |

Thank you for sharing your experiences! I appreciate your honesty so far. Finally, to wrap up, we are going to discuss a new type of treatment for HIV. In this treatment, instead of taking pills every day, you would receive two injections in your gluteal muscle or buttock once per month at the clinic. Once a person starts receiving the injections it is important that they come to the clinic every month, but, if they decide they don’t want to continue with the injections, they can go back to the daily pills. ART injections are not yet available for HIV treatment in most countries, but may become available soon.

1. Based on what we’ve just discussed, what are your initial thoughts or feelings about monthly injectable ART?

*Probes*: Is there anything appealing to you about it?

What concerns do you have about it?

Do you have any specific concerns as a person who injects drugs?

What questions would you want to ask about it?

1. Think about people you know and live in your community who inject drugs. In your opinion, what would they think about monthly injectable ART?

*Probes*: What would the find more appealing about it compared with daily oral ART?

What might be some drawbacks about it compared with daily oral ART?

1. How does it make you feel that the medication is injected into the muscle?

*Probes*: Do you have any physical concerns? (HINT: pain, swelling, infection)

1. How do you feel about coming in on a monthly basis for an injection?

*Probes:* How often do you visit the ART clinic now?

What kinds of challenges would there be to changing your routine?

1. Knowing what you now know, if you were given the choice of daily oral or monthly injectable ART, which would you prefer?

*Probes:* [IF YES/NO] What are a few main reasons you feel that way?

[IF UNSURE] What would help you decide?

[IF NO] Is there anything that would make you more amenable to monthly injectable ART?

**Closing Comments**: Do you have any further comments or questions? Thank you very much for sharing your experiences with me today! [MAKE REFERRALS AND OFFER ADDITIONAL RESOURCES AS APPROPRIATE]

**Interview Guide**

Feasibility and Acceptability of Long-Acting Injectable Antiretroviral Therapy (LAI ART) among HIV-Infected People who Inject Drugs (PWID) in Vietnam

*(VERSION: Pre-treatment ART-naïve PWID)*

**Purpose**: To characterize current access and adherence to daily oral ART and the feasibility and acceptability of long-acting injectable ART as an alternative.

**Population**: HIV-Infected PWID living in Hanoi or Khanh Hoa province.

**Introduction**: Hello and thank you for agreeing to speak with me today. My name is [NAME]. I am part of a research team seeking to improve treatment outcomes for people with HIV. Today we would like to ask you about your experience as a person with HIV. We would like to understand any previous experiences accessing or trying to access treatment for HIV. Finally, we would like to understand your thoughts and opinions about different treatment options, like taking a pill every day versus getting a monthly injection. I would like to learn about these things from your perspective.

Anything that you say to me will be kept confidential. What we discuss is audio recorded and then transcribed. When it is written out, all information that could identify you to someone reading the words (anything said with names or locations or that kind of thing) are completely removed. The material from our interview is saved with your identification number but not your name. The research team members reviewing this text will not link who you are to that ID and will not tell anyone on site about your specific feedback. There are other people doing interviews as well, and we will put all those documents together and look at main topics people talked about. We will not identify or share anything you say as a comment linked to your name or ID number.

Your input is crucial to help us improve ART access in Vietnam. It is important for you to know that there are no right or wrong answers. If there are any questions that make you feel uncomfortable, feel free to tell me and we can skip or come back to those questions.

You have signed the informed consent form in which you consented to audio record this discussion. I may also be taking notes on the things you are saying to help me follow the discussion. Please recall that when these tapes are not being reviewed, they will be stored in a locked location and will be destroyed after analyses are completed.

I would like to start recording. Before we do this, do you have any questions or concerns?

Are you ready to begin?

**Introduction**

To start off, I’d like to learn a little more about you.

1. Can you walk me through a typical day in your life?

*Probes*: What kinds of routines do you have? (HINT: work, school, medical appointments, meals)

How do you like to spend your free time?

Tell me about your job.

How long have you lived in this area?

1. Can you tell me about the important people in your life? This could be family, friends, people you work with – anyone who comes to mind.

*Probes*: Do you have family in this area?

Are you married? Have kids?

Who do you live with?

Who do you talk to when you need support, like a ride to the store?

What about people you use drugs with?

| RQ1. How do PWID currently describe accessing and adhering to ART? |
| --- |

Thank you for sharing with me. Next, I’d like to ask you a little bit about your experience living with HIV. I want to remind you again that everything you share is just between us. I won’t be sharing this information with your doctors or anyone else, so please feel free to talk honestly.

1. First, can you tell me how you came to learn that you have HIV?

*Probes*: Tell me all the things you remember about those few days.

Walk me through your diagnosis.

How did the medical staff treat you?

Did you disclose your status to anyone? Who did you tell first?

1. Have you ever discussed HIV treatment with a medical professional?

***IF NO, SKIP TO QUESTION 7.***

1. Think back to that encounter. Can you walk me through the conversation you had with the [doctor/medical professional]?

*Probes:* What did they tell you about treatment? (HINT: effectiveness, what happens to your body)

What did they tell you about the pills you would be taking? (HINT: how and when to take them, side effects, cost)

1. What kinds of things did you think about when you were deciding whether to start ART?

| RQ2. How do PWID describe the feasibility/acceptability of LAI ART, and how does this compare with their perceptions of daily oral ART? |
| --- |

Thank you for sharing your experiences! I appreciate your honesty so far. Next, I’d like to ask your opinions about two different treatment options for HIV. Just as a reminder, there are no right or wrong answers, so please feel comfortable sharing freely.

The questions that follow are going to discuss two types of treatment for HIV. Around the world, including here in Vietnam, persons with HIV are encouraged to take medications called “anti-retroviral therapy” or ART. ART is a type of medicine that fights HIV and can allow persons with HIV to live long, healthy lives. ART has to be taken for life – if someone stops taking the ART, the virus will no longer be under good control, and will eventually lead to worsening illness.

The first type of ART is an oral pill. People usually take 1 or 2 pills every day to keep the HIV virus under control in their bodies. The ART pills only work well in controlling HIV in someone’s body if they are taken every day. Once someone starts ART, they usually need to come back to clinic for ART refills every 3 or 6 months. The oral pill has some side effects for some people, such as nausea, vomiting, and upset stomach.

The second type of ART is a long-lasting injection. With this type of ART, instead of taking pills every day, the medication is delivered via two injections in your gluteal muscle or buttock once per month at the clinic. Each injection is about 2-3 milliliters. The injection includes anti-HIV drugs that remain in your body, fighting HIV infection, for an entire month. Unlike the pills, you would not have to take ART medication on a daily basis, but you would need to come back to the clinic once per month to get the injections. This type of injection has been studied by scientists in people with HIV. The injections are safe and effective. In research studies, people generally had similar side effects to those experienced with pills (such as nausea, vomiting, upset stomach) and the injections helped to fight HIV infection just as well as the daily pills. However, most people who received injections said that they had pain around where they received the shot. This pain usually went away in about 3 days. Once a person starts receiving the injections it is important that they come to the clinic every month, but, if they decide they don’t want to continue with the injections, they can go back to the daily pills. ART injections are not yet available for HIV treatment in most countries, but may become available soon.

1. Just to begin, can you tell me some similarities and differences between daily oral ART and monthly injectable ART?

*Probes:* What do you know about their effectiveness? (Are they similar? Different?)

What do you know about how they are taken or administered?

What do you know about how often they are taken or administered?

What do you know about how often you must visit the clinic?

What do you know about the side effects of each?

1. Let’s start with the first type of ART, daily oral pills. Based on what we’ve just discussed, what are your initial thoughts or feelings about daily oral pills?

*Probes*: Is there anything appealing to you about it?

What concerns do you have about it? (HINT: remembering to take medication, family members finding out)

Do you have any specific concerns as a person who injects drugs?

What questions would you want to ask about it?

1. And based on what we’ve discussed, what are your initial thoughts or feelings about monthly injectable ART?

*Probes*: Is there anything appealing to you about it?

What concerns do you have about it?

Do you have any specific concerns as a person who injects drugs?

What questions would you want to ask about it?

1. Think about people you know and live in your community who inject drugs. In your opinion, what would they think about monthly injectable ART?

*Probes*: What would the find more appealing about it compared with daily oral ART?

What might be some drawbacks about it compared with daily oral ART?

1. How does it make you feel that the medication is injected into the muscle?

*Probes*: Do you have any physical concerns? (HINT: pain, swelling, infection)

1. How do you feel about coming in on a monthly basis for an injection?

*Probes:* How often do you visit the ART clinic now?

What kinds of challenges would there be to changing your routine?

1. Knowing what you now know, if you were given the choice of daily oral or monthly injectable ART, which would you prefer?

*Probes:* [IF YES/NO] What are a few main reasons you feel that way?

[IF UNSURE] What would help you decide?

[IF NO] Is there anything that would make you more amenable to monthly injectable ART?

**Closing Comments**: Do you have any further comments or questions? Thank you very much for sharing your experiences with me today! [MAKE REFERRALS AND OFFER ADDITIONAL RESOURCES AS APPROPRIATE]

***Interview Guide***

*Feasibility and Acceptability of Long-Acting Injectable Antiretroviral Therapy (LAI ART) among HIV-Infected People who Inject Drugs (PWID) in Vietnam*

*(VERSION: PWID lost from ART care)*

***Purpose****: To characterize current access and adherence to daily oral ART and the feasibility and acceptability of long-acting injectable ART as an alternative.*

***Population****: HIV-Infected PWID living in Hanoi or Khanh Hoa province.*

***Introduction****: Hello and thank you for agreeing to speak with me today. My name is [NAME]. I am part of a research team seeking to improve treatment outcomes for people with HIV. As you may know, ART has traditionally been available from clinics as a daily oral tablet. More recently, scientists have developed an alternative form of ART available as a monthly injection. Research indicates that the monthly injection is as effective as the daily oral formulation in maintaining HIV viral suppression.*

*Today we would like to ask you about your experience as a person with HIV. We would like to understand your previous experiences accessing or trying to access treatment for HIV. We would also like to understand your thoughts and opinions about current treatment options. Finally, we would like to ask you your opinions about the monthly injectable form of ART. I would like to learn about these things from your perspective.*

*Anything that you say to me will be kept confidential. What we discuss is audio recorded and then transcribed. When it is written out, all information that could identify you to someone reading the words (anything said with names or locations or that kind of thing) are completely removed. The material from our interview is saved with your identification number but not your name. The research team members reviewing this text will not link who you are to that ID and will not tell anyone on site about your specific feedback. There are other people doing interviews as well, and we will put all those documents together and look at main topics people talked about. We will not identify or share anything you say as a comment linked to your name or ID number.*

*Your input is crucial to help us improve ART access in Vietnam. It is important for you to know that there are no right or wrong answers. If there are any questions that make you feel uncomfortable, feel free to tell me and we can skip or come back to those questions.*

*You have signed the informed consent form in which you consented to audio record this discussion. I may also be taking notes on the things you are saying to help me follow the discussion. Please recall that when these tapes are not being reviewed, they will be stored in a locked location and will be destroyed after analyses are completed.*

*I would like to start recording. Before we do this, do you have any questions or concerns?*

*Are you ready to begin?*

***Introduction***

*To start off, I’d like to learn a little more about you.*

1. *Can you walk me through a typical day in your life?*

*Probes: What kinds of routines do you have? (HINT: work, school, medical appointments, meals)*

*How do you like to spend your free time?*

*Tell me about your job.*

*How long have you lived in this area?*

1. *Can you tell me about the important people in your life? This could be family, friends, people you work with – anyone who comes to mind.*

*Probes: Do you have family in this area?*

*Are you married? Have kids?*

*Who do you live with?*

*Who do you talk to when you need support, like a ride to the store?*

*What about people you use drugs with?*

| *RQ1. How do PWID currently describe accessing and adhering to ART?* |
| --- |

*Thank you for sharing with me. Next, I’d like to ask you a little bit about your experience living with HIV. I want to remind you again that everything you share is just between us. I won’t be sharing this information with your doctors or anyone else, so please feel free to talk honestly.*

1. *First, can you tell me how you came to learn that you have HIV?*

*Probes: Tell me all the things you remember about those few days.*

*Walk me through your diagnosis.*

*How did the medical staff treat you?*

*Did you disclose your status to anyone? Who did you tell first?*

1. *Can you walk me through the process of being on ART?*

*Probes: About how long ago did you get off of ART?*

*And when you were on ART, about how long were you on it?*

*How did you decide to begin ART? Did anyone help you make this decision?*

*What did you see as the pros and cons of being on ART?*

*How did you feel about beginning ART? (HINT: Hopeful? Scared?)*

*How did you feel when you stopped treatment?*

1. *Can you tell me about anything that made it difficult to stay engaged in ART?*

*Probes: Can you describe any times when you…*

*… Forgot to take your medicine?*

*… Didn’t have your medicine on hand, for example, because you were out of the home?*

*… Didn’t feel like you wanted to take your medicine? (What made you feel that way?)*

*… Stopped your treatment altogether, like for months or longer? How often did it happen?*

*Can you think of anything that would have made it easier to take your medicine?*

1. *For many people, injection drug use can get in the way of day-to-day living. Can you think of any ways that your drug use made it harder to take ART daily? (HINT: community/provider stigma, low motivation, lack of knowledge about ART, transportation/access/money issues)*

*Probes: …when you traveled to the clinic?*

*…when you were talking to medical staff?*

*…when you were at home with your family?*

| *RQ2. How do PWID describe the feasibility/acceptability of LAI ART, and how does this compare with their perceptions of daily oral ART?* |
| --- |

*Thank you for sharing your experiences! I appreciate your honesty so far. Finally, to wrap up, we are going to discuss a new type of treatment for HIV. In this treatment, instead of taking pills every day, you would receive two injections in your gluteal muscle or buttock once per month at the clinic. Once a person starts receiving the injections it is important that they come to the clinic every month, but, if they decide they don’t want to continue with the injections, they can go back to the daily pills. ART injections are not yet available for HIV treatment in most countries, but may become available soon.*

1. *Based on what we’ve just discussed, what are your initial thoughts or feelings about monthly injectable ART?*

*Probes: Is there anything appealing to you about it?*

*What concerns do you have about it?*

*Do you have any specific concerns as a person who injects drugs?*

*What questions would you want to ask about it?*

1. *Think about people you know and live in your community who inject drugs. In your opinion, what would they think about monthly injectable ART?*

*Probes: What would the find more appealing about it compared with daily oral ART?*

*What might be some drawbacks about it compared with daily oral ART?*

1. *How does it make you feel that the medication is injected into the muscle?*

*Probes: Do you have any physical concerns? (HINT: pain, swelling, infection)*

1. *How do you feel about coming in on a monthly basis for an injection?*

*Probes: How often did you visit the clinic when you were on ART?*

1. *Since stopping ART, have you given any thought to returning to treatment?*

*Probes: Can you tell me why you feel that way?*

*If monthly injectable ART were available to you, how would this impact your thoughts on returning to treatment?*

1. *Knowing what you now know, if you were given the choice of daily oral or monthly injectable ART, which would you prefer?*

*Probes: [IF YES/NO] What are a few main reasons you feel that way?*

*[IF UNSURE] What would help you decide?*

*[IF NO] Is there anything that would make you more amenable to monthly injectable ART?*

***Closing Comments****: Do you have any further comments or questions? Thank you very much for sharing your experiences with me today! [MAKE REFERRALS AND OFFER ADDITIONAL RESOURCES AS APPROPRIATE]*

**Interview Guide**

Feasibility and Acceptability of Long-Acting Injectable Antiretroviral Therapy (LAI ART) among HIV-Infected People who Inject Drugs (PWID) in Vietnam

*(VERSION: Stakeholders)*

**Purpose**: To characterize current access and adherence to daily oral ART and the feasibility and acceptability of long-acting injectable ART as an alternative.

**Population**: HIV stakeholders (e.g., Vietnam Authority for HIV/AIDS Control, the Director of the provincial Centers for Disease Control) in Vietnam.

**Introduction**: Hello and thank you for agreeing to speak with me today. My name is [NAME]. I am part of a research team seeking to improve treatment outcomes for people with HIV. As you may know, ART has traditionally been available as a daily oral tablet. More recently, scientists have developed a long-acting injectable form of ART. Research indicates that the monthly injection is as effective as the daily oral formulation in maintaining HIV viral suppression.

Today we would like to have a conversation with you about monthly injectable ART. We would like to understand your current work as a health authority working with HIV-positive populations. We would like to understand your thoughts and opinions about current treatment options. Finally, we would like to ask you your opinions about the monthly injectable form of ART, and whether you think this would be a good option for people with HIV in Vietnam. I would like to learn about these things from your perspective. It is important for you to know that there are no right or wrong answers. If there are any questions that make you feel uncomfortable, feel free to tell me and we can skip or come back to those questions. Do you have any questions before we begin?

**Introduction**

To start off, I’d like to learn a little more about your professional role.

1. Can you please tell me about your role here at [ORGANIZATION NAME]?

*Probes:* Walk me through a day at work.

What are your main responsibilities?

1. What kinds of policies or projects do you work on that involve ART?

*Probes*: What types of organizations do you interact with on a regular basis? (HINT: ART clinic directors, methadone clinic directors, hospitals, other government agencies)

What do these interactions look like?

| RQ1. How do stakeholders currently describe the state of ART delivery in Vietnam? |
| --- |

Thank you for sharing with me. Next, I’d like to ask you a little bit about the current state of ART in Vietnam. We’re especially interested in access and adherence to ART among people with HIV. We’d like to learn a little more about your experiences and opinions about this.

1. As you may know, about 65% of people with HIV are in treatment. What are some of the main challenges or barriers to increasing treatment rates for people with HIV?
2. We are especially interested in ART access and adherence for people who inject drugs. What are some additional challenges or barriers to improve treatment in this population?

*Probes:* Are you familiar with any programs that are intended to improve access or adherence specifically among people who inject drugs?

1. What current policies or strategies do you know of that prioritize people with HIV who are lost to care?

*Probes:* Can you discuss how any of these strategies engage people who inject drugs?

1. In your opinion, what are some major policy priorities for improving ART uptake in Vietnam?

*Probes*: Can you think of any priorities that are especially important for people who inject drugs?

| RQ2. How do stakeholders describe the feasibility/acceptability of LAI ART, and how does this compare with their perceptions of daily oral ART? |
| --- |

Thank you for sharing your experiences! I appreciate your honesty so far. Next, I’d like to ask you your opinions about a new type of ART that doesn’t need to be taken on a daily basis.

1. Before today, had you ever heard of injectable ART?

*Probes:* [IF YES] What do you know about it?

[IF YES] And where did you hear about it?

The questions that follow are going to discuss a new type of treatment for HIV. With this treatment, instead of a person with HIV taking pills every day to control the virus, they receive an injection every month. You may have heard of this type of injectable ART for HIV pre-exposure prophylaxis – what we are discussing here is the injectable ART used for treatment of HIV for people with known HIV infection, not prevention. Much like oral ART, the injection includes two different kinds of anti-HIV drugs (two separate injections, into the gluteal muscle or buttock of the patient). According to the two Phase 3 randomized controlled studies comparing oral ART to the injectable ART, the once-monthly injectable ART performed as well as the oral drugs in maintaining HIV viral suppression. The injectable ART remains active in the body for at least 4-weeks (1 month). Again looking at the results from the clinical studies, there was no difference between the oral ART and injectable ART in terms of safety or side effects. Once a patient starts receiving the injections it is important that they come to the clinic every month, but, if they decide they don’t want to continue with the injections, they can go back to the daily oral ART. ART injections are not yet available for HIV treatment in most countries, but may become available soon.

1. Based on what we’ve just discussed, what is your initial reaction to monthly injectable ART?

*Probes*: Can you tell me anything that is appealing to you about it?

What questions or concerns do you have about it?

How, if at all, might LAI ART address some of the barriers and challenges you mentioned before?

1. In your opinion, how acceptable would injectable ART be to clinics?

*Probes:* How would clinic directors or providers respond to injectable ART becoming available?

What kinds of changes would need to happen in clinics for the transition to be successful? (HINT: new protocols, training staff, changing roles, space/facilities)

1. The research on injectable ART is promising, but it is still new and not yet available in most countries. In your opinion, is Vietnam a suitable candidate as an early adopter of this technology?

*Probes:* Tell me why you feel that way.

1. The cost of injectable ART versus daily oral ART is currently unknown. How do you think cost would impact the feasibility of introducing injectable ART on a large scale?

*Probes:* Besides the price of the medication, what might be some other costs of introducing injectables into ART clinics? (HINT: programmatic expenses, staff training, increased number of clinic visits, costs of reengaging clients lost from care)

1. From a policy standpoint, what steps would need to be taken for a new medication like injectable ART to be introduced into Vietnam’s healthy system? (HINT: Any approval processes, DAV market authorization, SHI reimbursements, commodity procurement).

*Probes:* What government policies might be potential barriers to the introduction of injectable ART into Vietnam’s health system?

What policies might *facilitate* introduction of injectable ART into Vietnam’s health system?

Think about the current transition in Vietnam from TLE to TLD* as a first-line regimen for HIV treatment. Are there implementation challenges that might be the same? *(TLE: Tenofovir/Lamivudine/Efavirenz, TLD: Tenofovir/Lamivudine/Dolutegravir)*

1. Other than your own organization, what organizations would be most important to the success of introducing injectable ART in Vietnam? (HINT: MOH/VAAC, provincial departments of health, PEPFAR, GFATM, USAID, UNAIDS, Vietnam CDC, Drug Authority of Vietnam)

*Probes:* What role would that organization play?

1. Thank you for your answers so far. I have one final question. Can you tell me one thing that excites you and one thing that concerns you about introducing injectable ART into clinics in Vietnam?

**Closing Comments**: Do you have any further comments or questions? Thank you very much for sharing your experiences with me today.

**Interview Guide**

Feasibility and Acceptability of Long-Acting Injectable Antiretroviral Therapy (LAI ART) among HIV-Infected People who Inject Drugs (PWID) in Vietnam

*(VERSION: Clinicians)*

**Purpose**: To characterize current access and adherence to daily oral ART and the feasibility and acceptability of long-acting injectable ART as an alternative.

**Population**: ART clinic staff (e.g., doctors, pharmacists, counselors, directors) in Hanoi or Khanh Hoa province.

**Introduction**: Hello and thank you for agreeing to speak with me today. My name is [NAME]. I am part of a research team seeking to improve treatment outcomes for people with HIV. As you are aware, ART has traditionally been available as a daily oral tablet. More recently, scientists have developed an alternative form of ART available as a monthly injection. Research indicates that the monthly injection is as effective as the daily oral formulation in maintaining HIV viral suppression.

Today we would like to have a conversation with you about monthly injectable ART. We would like to understand your current work providing ART to people with HIV. We would like to understand your thoughts and opinions about current treatment options. Finally, we would like to ask you your opinions about the monthly injectable form of ART, and whether you think this would be a good option for your clients and for your clinic. I would like to learn about these things from your perspective. It is important for you to know that there are no right or wrong answers. If there are any questions that make you feel uncomfortable, feel free to tell me and we can skip or come back to those questions. Do you have any questions before we begin?

**Introduction**

To start off, I’d like to learn a little more about your professional role.

1. Can you please tell me about your role here at the clinic?

*Probes:* Walk me through a day at work.

Who do you interact with on a daily basis? (HINT: other staff, supervisors, clients)

What do your interactions with other staff look like? How do you work together?

What are your main responsibilities?

How long have you been in this position?

1. Can you tell me about your relationship with the ART clients here?

*Probes*: How many clients do you interact with in a day?

Walk me through a typical client visit.

Can you give me an example of a recent interaction with a client?

How are patients typically counseled about adherence?
 How is adherence assessed?

How often do clients receive a viral load test, and how does this impact counseling?

| RQ1. How do clinic staff currently describe PWID access and adherence to ART? |
| --- |

Thank you for sharing with me. Next, I’d like to ask you a little bit about your clients. We’re especially interested in people who inject drugs. We’d like to learn a little more about your experiences and opinions about this.

1. What kinds of barriers or challenges do people who inject drugs face when starting ART? (HINT: cost of care, fear of doctors, stigma)

*Probes:* How do these barriers differ from the barriers that your other clients face?

Imagine a PWID walks into the clinic. How would you counsel them about starting ART?

How is your advice typically received by PWID? (If you can recall one, please give me an example of a recent conversation you had with a person who uses drugs)

1. And what kinds of barriers or challenges do people who inject drugs face when staying on ART long-term? (HINT: showing up for appointments, remembering to take medicine, stigma)

*Probes:* How do these barriers differ from the barriers that your other clients face? Do you notice any patterns?

Imagine a PWID is struggling to adhere to ART. How would you counsel them?

How is your advice typically received by PWID? (If you can recall one, please give me an example of a recent conversation you had with a person who injects drugs)

| RQ2. How do clinic staff describe the feasibility/acceptability of LAI ART, and how does this compare with their perceptions of daily oral ART? |
| --- |

Thank you for sharing your experiences! I appreciate your honesty so far. Finally, I’d like to ask you your opinions about a new type of ART that doesn’t need to be taken on a daily basis.

1. Before today, had you ever heard of injectable ART?

*Probes:* [IF YES] What do you know about it?

[IF YES] And where did you hear about it?

The questions that follow are going to discuss a new type of treatment for HIV. With this treatment, instead of a person with HIV taking pills every day to control the virus, they receive an injection every month. You may have heard of this type of injectable ART for HIV pre-exposure prophylaxis – what we are discussing here is the injectable ART used for treatment of HIV for people with known HIV infection, not prevention. Once a patient starts receiving the injections it is important that they come to the clinic every month, but, if they decide they don’t want to continue with the injections, they can go back to the daily oral ART. ART injections are not yet available for HIV treatment in most countries, but may become available soon.

1. Based on what we’ve just discussed, what is your initial reaction to monthly injectable ART?

*Probes*: Can you tell me anything that is appealing to you about it?

What questions or concerns do you have about it?

Do you have any specific concerns about monthly injectable ART for your clients who inject drugs?

1. Imagine your clinic were to offer the option of daily oral or monthly injectable ART to your clients. How would you, personally, feel about this change?

*Probes*: How would you decide when to offer daily oral? Long acting injectable?

How would you feel about clients having the option of choosing?

What kinds of clients might injectable ART be most appropriate for?
 What kinds of challenges for clients might injectable ART address?

1. When you imagine incorporating monthly injectable ART into the clinic’s offerings, what might be some challenges for your clinic as a whole?

*Probes:* What kinds of changes would need to happen in the clinic for the transition to be successful? (HINT: new protocols, training staff, changing roles, space/facilities)

Can you think of any other times your clinic started a new medicine or technology? What kinds of changes happened and how did you respond?

What do you think your colleagues would think about monthly ART?

1. When you imagine incorporating monthly injectable ART into your own daily practice, what might be some professional challenges you face as a (clinician/counselor/pharmacist/clinic director)?

*Probes*: In what ways would your job be easier?

In what ways would your job be more difficult?

How do you imagine your day-to-day job might change?

What are your thoughts about…

…Patient visits increasing in frequency?

…Adjusting to new procedures (e.g., training staff on injecting)?

…Ordering or storing new medications and supplies?

…Counseling patients on new side effects (e.g., injection site swelling)?

1. Thinking specifically about your clients who inject drugs and are currently on ART, how might they feel about monthly injectable as an alternative to the daily oral tablet?

*Probes:* How appealing do you think it would be to your clients?

How willing would the average client be to switch?

What might they be hesitant about?

How would you counsel them?

1. Now, let’s continue thinking about people who inject drugs. Imagine a new client comes into the clinic who has never used ART, or who used it in the past but stopped. How do you think they might feel if you told them about monthly injectable ART?

*Probes:* How attractive would a monthly injection be to a new client when compared with a daily oral tablet?

1. Thank you for your answers so far. I have one final question. Can you tell me one thing that excites you and one thing that concerns you about incorporating injectable ART into your clinic?

**Closing Comments**: Do you have any further comments or questions? Thank you very much for sharing your experiences with me today!
